# Griseaketides A–D, New Aromatic Polyketides from the Pathogenic Fungus *Magnaporthe grisea*

**DOI:** 10.3390/molecules25010072

**Published:** 2019-12-24

**Authors:** Yin-He Yang, Da-Song Yang, Hong-Mei Lei, Cheng-Yun Li, Guo-Hong Li, Pei-Ji Zhao

**Affiliations:** 1 State Key Laboratory for Conservation and Utilization of Bio-Resources in Yunnan, Yunnan University, Kunming 650091, China; 2 Institute of Entomoceutics Research, Dali University, Dali 671000, China; 3 State Key Laboratory for Conservation and Utilization of Bio-Resources in Yunnan, Yunnan Agricultural University, Kunming 650205, China

**Keywords:** *Magnaporthe grisea*, aromatic polyketide, rice blast disease, nematicidal activity

## Abstract

*Magnaporthe grisea* is the causal agent of rice blast disease, which is the most serious disease of cultivated rice. Aromatic polyketides are its typical metabolites and are involved in the infection process. In the search for novel lead compounds, chemical investigation of the fungus *M. grisea* M639 has led to the isolation of four new aromatic polyketides (salicylaldehyde skeleton bearing an unsaturated side chain), griseaketides A–D (**1**–**4**), as well as 15 known compounds (**5**–**19**). The structures of the new compounds were elucidated on the basis of extensive spectroscopic analyses, including HR-MS, 2D NMR. Compound **12** showed prominent activity that killed 94.5% of *C. elegans* at 400 ppm and 66.9% at 200 ppm over 24 h. This is the first report describing the nematicidal activity of this type aromatic polyketide.

## 1. Introduction

Exploration of natural sources of novel bioactive compounds as drugs or lead compounds has been an emerging field over the past decades, and exciting evidence has been provided by the isolation of microbe-derived metabolites [1]. The fungal kingdom includes many species with unique and unusual biochemical pathways, which results in lots of structurally fascinating secondary metabolites with promising biological and pharmacological properties, such as penicillin, cyclosporine and statins [2].

A group of structurally and actively diverse metabolites were produced by plant pathogenic strains [3,4,5], which are responsible for function as essential determinants of pathogenicity or virulence. The rice blast fungus *Magnaporthe grisea* (imperfect stage of *Pyricularia grisea* Sacc.) causes a serious disease on agriculturally significant plants including rice, wheat, and barley. Each year rice blast causes losses of 10–30% of the rice harvest and therefore poses a threat to the world’s most important food security crop [6]. Previous, studies have shown that aromatic polyketide phytotoxins [7], *O*-nitrophenol derivatives [8], and naphthalenones [9] are the typical secondary metabolites of *M. grisea*. Some secondary metabolites (such as melanin, siderophores and fumonisin) of pathogenic fungi often function as essential determinants of pathogenicity and are involved in the infection process [10,11]. Thus, examining their secondary metabolism leads to the discovery of novel compounds with interesting structures or modes of action that could be useful for agrochemistry or pharmacology [5]. In the work, chemical investigations of a strain *M. grisea* M639 have led to the discovery of four new aromatic polyketides griseaketides A–D (**1**–**4**), as well as 15 known compounds (**5**–**19**). Herein, we describe the isolation, structural elucidation and nematicidal activities of these compounds.

## 2. Results

**Figure 1:**
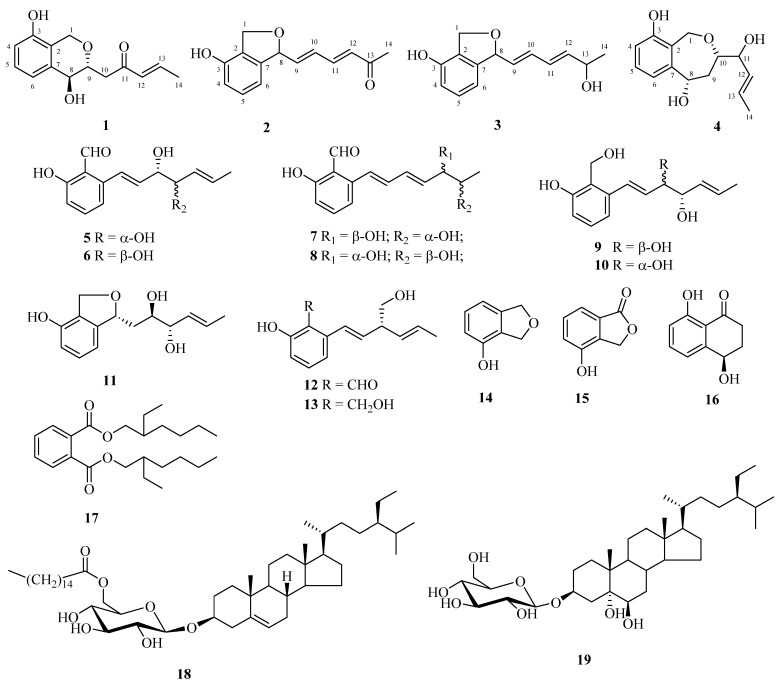
Structures of compounds **1**−**19**.

**Figure 2:**
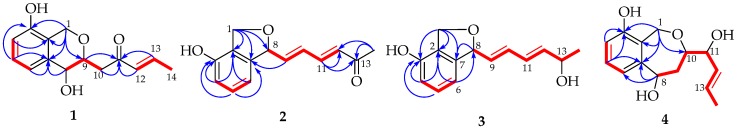
Selected HMBC (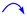) and ^1^H-^1^H COSY (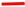) correlations of compounds **1**–**4**.

Griseaketide A (**1**) was isolated as a pale yellow amorphous powder. Its molecular formula C_14_H_16_O_4_ was determined by HR-ESI-MS and ^13^C NMR coupled with DEPT spectra, which indicated 7 degrees of unsaturation (Table 1). The ^1^H NMR spectrum exhibited signals for one methyl resonance at *δ*_H _1.94 (3H, dd, *J* = 6.8, 1.4 Hz), three aromatic protons at *δ*_H_ 6.65 (1H, d, *J* = 8.7 Hz), 7.11 (1H, t, *J* = 7.5 Hz) and 7.12 (1H, d, *J* = 7.5 Hz), two olefinic protons at *δ*_H_ 6.24 (1H, dd, *J* = 15.8, 1.4 Hz) and 6.99 (1H, dq, *J* = 15.8, 6.8 Hz) and four monooxygenated protons at *δ*_H_ 3.93 (1H, m), 4.49 (1H, d, *J* = 7.5 Hz), 4.85 (1H, d, *J* = 15.8 Hz), 4.70 (1H, d, *J* = 15.8 Hz). Sixteen carbon resonances observed in the ^13^C NMR spectrum coupled with the DEPT interpretations of **1** were attributable to one ketone (*δ*_C_ 199.2), one pair of disubstituted double bonds at *δ*_C_ 132.2 (d) and *δ*_C _144.6 (d), one monooxygenated 1,2,3-trisubstituted aromatic ring [*δ*_C_ 150.8 (s), 138.3 (s), 121.9 (s), 113.7 (d), 127.7 (d), 118.4 (d)], one primary alcohol at *δ*_C_ 64.3 (t), two oxymethine groups at *δ*_C_ 69.2 (d) and 75.4 (d), and one methyl group at *δ*_C_ 18.4 (q). On the basis of spectroscopic data analysis, compound **1** was deduced as an aromatic polyketide (Figure 1), similar to 3-(1’,3’-pentadienyl)-3,4-dihydro-l*H*-2-benzopyran-4,8-diol [7]. A preliminary linear skeleton bearing two branches was deduced to be −C-8−C-9−C-10 (−branch) −C-12−C-13−C-14 (−branch) from complete interpretations of key cross-peaks in the COSY spectrum (H-8/H-9/H-10; H-12/H-13/H-14) together with key cross-peaks in the HMBC spectrum (Figure 2): from H-8 to C-9 and C-10; from H-10 to C-8, C-9 and C-11; from H-14 to C-12 and C-13. The HMBC correlation of H-1/C-9 demonstrated that the formation of an extra pyran ring was through a bridge between C-1 and C-9. Typically, values of ^3^J ≤ 2 Hz suggest *cis* configuration, and ^3^J ≥5 Hz suggest *trans* configuration [12], so the *trans*-configuration was assigned to H-8 and H-9 on the basis of its larger coupling constant (7.5 Hz). As a result, the relative configuration of compound **1** is shown in Figure 1 and named as griseaketide A.

Compound **2** was obtained as optically active amorphous powder (${\left[\alpha \right]}_{D}^{18}$ –16.0 *c* 0.16, MeOH) and its molecular formula of C_14_H_14_O_3_ was established by the negative HR-ESI-MS, revealing 8 degrees of unsaturation. Analysis of the ^1^H and ^13^C NMR (Table 1) data of **2** revealed the presence of a monooxygenated 1,2,3-trisubstituted aromatic ring, two pairs of double bonds, a primary alcohol, a carbonyl group, an oxymethine and one methyl (Figure 1). These signals revealed that **2** is similar to **1** and suggested to be an aromatic polyketide [13]. The lower-field shifting of carbon signal (*δ*_C-8_ 84.4) in **2** and the observed HMBC correlations (Figure 2) from H-1 to C-8 suggested that C-1 and C-8 are adjacent substituents on the aromatic ring and form a 1,3-dihydroisobenzofuran system. One branch was deduced to be −C-8−C-9−C-10−C-11−C-12 from complete interpretations of key cross-peaks in the COSY spectrum (H-8/H-9/H-10/H-11/H-12) together with key cross-peaks (from H-8 to C-9 and C-10; from H-10 to C-8, C-11 and C-12; from H-11 to C-9, C-10 and C-13; from H-14 to C-12 and C-13) in the HMBC spectrum to determinate the side chain moiety (Figure 2). The configuration of conjugated double bonds was elucidated as *E* geometries by their coupling constants (*J*_9−10_ = 15.1 Hz, *J*_11−12_ = 15.7 Hz) [14]. The configurations of C-8 cannot be determined by the present data. Thus, the structure of **2** was determined as shown in Figure 1 and named as griseaketide B. 

Compound **3** was obtained as a pale yellow oil. HR-ESI-MS analysis of **3** provided a molecular formula of C_14_H_16_O_3_, corresponding to an unsaturation number of 6. The UV absorption maxima at 226 and 271 nm suggested the presence of a conjugated system in the molecule. Compound **3** was very similar to compound **2**, but the ketone (*δ*_C_ 198.8) of **2** was replaced by one hydroxyl in **3** (Table 2). The ^1^H−^1^H COSY spectrum of compound **3** revealed two structural fragments to be −C-8−C-9−C-10−C-11−C-12−C-13−C-14 and −C-4−C-5−C-6− by the clear correlations of H-8/H-9/H-10/H-11/H-12/H-13/H-14 and H-4/H-5/H-6 (Figure 2). The HMBC experiment showed correlations from H-1 to C-8 to suggest that C-1 and C-8 are adjacent substituents on the aromatic ring and formed a 1,3-dihydroisobenzofuran system (Figure 2). The *E* geometries of the two double bonds was inferred by coupling constants (*J*_9−10_ = 15.1 Hz, *J*_11−12_ = 15.2 Hz) [14]. The configurations of C-8 and 13-OH cannot be determined by the present data. Thus, the relative configuration of **3** is shown as Figure 1 and named as griseaketide C.

**Figure 3:**
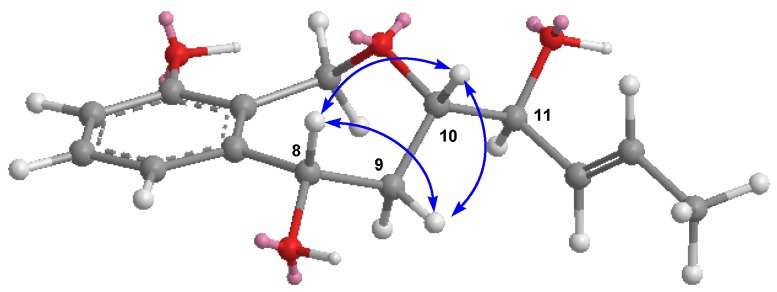
Key ROESY correlations of compound **4**.

The molecular formula of griseaketide D (**4**) was found to be C_14_H_18_O_4_ by HR-ESI-MS and ^13^C NMR analysis. Its ^1^H and ^13^C NMR (Table 2) signals were similar to that of **1**, except for the carbonyl group in **1** that was replaced by a hydroxyl group at C-11. On the basis of the consecutive COSY correlations from H-8 to H-14 (Figure 2) and the coupling constants (*J*_12−13_ = 14.6 Hz), the side chain was elucidated as 1’,3’,4’-trihydroxy-5’*E*-heptaenyl. The observed HMBC (Figure 2) correlations of H-1/C-10 suggested a seven membered ether ring was fused to the trisubstituted benzene ring through an oxygen bridge between C-1 and C-10. The relative configuration of **4** was deduced from ROESY correlations (Figure 3) and comparisons with data reported in the literature [15]. The ROESY cross-peaks of H-8/H-9β and H-10 indicated that they are all cofacial and assigned as β-oriented which were consistent with xylarinol B [15]. The configuration of 11-OH cannot be determined by the present data. Accordingly, the structure and relative configuration of **4** was established as shown.

The known compounds were identified as (1’*E*,3’*S*,4’*S*,5’*E*)-2-(3’,4’-dihydroxy-1’,5’-heptadienyl)-6-hydroxybenzaldehyde (**5**) [16], (1’*E*,3’*S*,4’*R*,5’*E*)-2-(3’,4’-dihydroxy-1’,5’-heptadienyl)-6-hydroxy benzaldehyde (**6**) [17], (5’*R*,6’*S*)-pyriculariol (**7**) [18], (5’*S*,6’*R*)-pyriculariol (**8**) [18], dihydropyriculol (**9**) [7], *epi*-dihydropyriculol (**10**) [7], pyriculin A (**11**) [19], (*R*)-pyricuol (**12**) [20], (1’*E*,3’*R*,4’*E*)-2- hydroxymethyl-3-(3’-hydroxymethylhexa-1’,4’-dienyl)phenol (**13**) [21], 1,3-dihydro-4-isobenzo- furanol (**14**) [22], 4-hydroxyphthalide (**15**) [23], (−)-isosclerone (**16**) [24], bis (2-ethylhexyl) phthalate (**17**) [25], daucosterol palmitate (**18**) [26] and 5*α*,6*β*-dihydroxydaucosterol (**19**) [27] by comparison of their experimental and reported spectroscopic data.

**Nematicidal activityof compounds.** The pure compounds **1** and **5**–**12** were tested for their nematicidal activity. Compounds **5**–**8** showed weak nematicidal activities against *Caenorhabditis elegans* at 400 ppm over 48 h, but compound **12** showed prominent activity that killed 94.5% of *C. elegans* at 400 ppm and 66.9% at 200 ppm over 24 h. 

## 3. Discussion

Rice blast, caused by infection of the rice blast fungus, *Magnaporthe grisea*, is the most destructive pathogen of rice worldwide. Many metabolites from rice blast fungus have been identified and they show different activities. Pyriculol caused a necrotic lesion in a rice wounding assay and showed inhibition in a spore germination bioassay [28]. Compound pyricuol inhibited shoot growth showing a stronger effect than pyriculol and dihydropyriculol [28,29]. In our experiment, part salicylaldehyde-type products showed nematicidal activity, and among them aldehyde group-containing compounds showed nematicidal activity, which is consistent with the literature [30], while pyricuol (**12**) showed a prominent nematicidal activity, which was distinguished by a different substitute on the side chain, and this further provides us with an indication of the nematicidal active compounds.

## 4. Materials and Methods

### 4.1. General Experimental Procedures

The optical rotations were measured with a Horiba SEPA-300 polarimeter (Kyoto, Japan). UV spectra were recorded on a Shimadzu UV-2401PC spectrophotometer (Kyoto, Japan). The NMR spectra were obtained with an Avance III 600 spectrometer (Bruker Biospin, Rheinstetten, Germany). The ESI and HR-ESI-MS were recorded on a Finnigan LCQ-Advantage (Thermo Finnigan, San Jose, CA, USA) and VG Auto-Spec-3000 mass spectrometer (VG, Manchester, England), respectively. Column chromatography (CC) was performed on Sephadex LH-20 (Amersham Biosciences, Piscataway, NJ, USA), silica gel (200–300 mesh, Qingdao Marine Chemical Inc., Qingdao, China), and RP-18 gel (LiChroprep, 40–63 μm; Merck, Darmstadt, Germany). Semipreparative HPLC was performed on an LC3000 (Beijing Chuangxintongheng Science & Technology Co., Ltd., Beijing, China). Fractions were monitored by TLC and visualized by heating plates sprayed with 5% H_2_SO_4_ in EtOH. 

### 4.2. Microbial Material 

The fungal strain of *Magnaporthe grisea* M639 used in this study was isolated from the leaf spot lesions of rice collected from Yunnan province, China, in August 2012. The strain has been preserved in the State Key Laboratory for Conservation and Utilization of Bio-Resources in Yunnan, Yunnan University.

### 4.3. Cultivation, Extraction and Isolation 

The strain* M. grisea* M639 was cultured on PDA solid medium at 26 °C for 7 days, and then it was inoculated into 1 L Erlenmeyer flasks each containing 200 mL of sticky rice-glucose liquid medium, which were cultivated at 26 °C for 14 days. The obtained culture filtrates were extracted by EtOAc three times to give a crude extract (1.32 g). The residual H_2_O portion was extracted with *n*-butyl alcohol to yield a residue (14.11 g). The EtOAc fraction was separated by CC on RP-18 (MeOH–H_2_O, 1:4 to 1:0) to produce six fractions (Fr.1–Fr.6). Fr.1 was purified by Sephadex LH-20 (CHCl_3_–MeOH, 1:1) and then chromatographed over a silica gel CC (CHCl_3_–MeOH, 100:1 to 2:1) to yield **14** (4.2 mg) and **15** (14.4 mg). Fr.2 was repeatedly subjected to silica gel CC eluting with CHCl_3_–MeOH (50:1 to 50:3) and then purified by Sephadex LH–20 CC (CHCl_3_–MeOH, 1:1) to afford **1** (5.7 mg), **9** (3.2 mg), **10** (7.2 mg), **11** (26.0 mg) and **16** (1.2 mg). Fr.3 was subjected to Sephadex LH-20 (MeOH) and then purified by silica gel to yield **4** (2.1 mg). Fr.4 was repeatedly purified by Sephadex LH-20 CC (CHCl_3_–MeOH, 1:1), semipreparative RP-18 CC (MeOH–H_2_O, 1:1) and silica gel CC (CHCl_3_–MeOH, 50:1) to obtain **3** (4.0 mg), **2** (1.8 mg), **5** (66.0 mg), **6** (6.5 mg), **7** (18.5 mg), **8** (5.7 mg) and **13** (1.1 mg). Fr.5 was subjected to Sephadex LH-20 CC (CHCl_3_–MeOH, 1:1), followed by semipreparative RP-18 CC (MeOH–H_2_O, 65:35) to yield **12** (7.0 mg). Fr.6 was chromatographed over a silica gel CC (petroleum ether–EtOAc, 25:1 to 3:2) and then purified by Sephadex LH-20 CC (CHCl_3_–MeOH, 1:1) to obtain **18** (6.7 mg) and **19** (1.8 mg). The *n*-butyl alcohol portion was chromatographed over silica gel CC (CHCl_3_–MeOH, 9:1 to 0:1) and then purified by Sephadex LH-20 CC (CHCl_3_–MeOH, 1:1) to give **17** (0.7 mg).

*Griseaketide A (**1**)*: Pale yellow amorphous powder; ${\left[\alpha \right]}_{D}^{18}$ –29.1 (*c* 0.15, MeOH); UV (MeOH) *λ*_max_ (log *ε*) 202 (4.53), 220 (4.34), 274 (3.43) nm; ^1^H NMR (CDCl_3_, 600 MHz) and ^13^C NMR (CDCl_3_, 150 MHz) data, see Table 1; positive ESI-MS *m*/*z* 271 [M + Na]^+^, 287 [M + K]^+^, 519 [2M + Na]^+^, 535 [2M + K]^+^; HR-ESI-MS *m*/*z* 247.0978 [M − H]^−^ (calcd for C_14_H_15_O_4_, 247.0976).

*Griseaketide B (**2**)*: Colorless amorphous powder; ${\left[\alpha \right]}_{D}^{18}$ –16.0 (*c* 0.16, MeOH); UV (MeOH) *λ*_max_ (log *ε*) 202 (4.23), 270 (3.90) nm; ^1^H NMR (CDCl_3_, 600 MHz) and ^13^C NMR (CDCl_3_, 150 MHz) data, see Table 1; negative ESI-MS *m*/*z* 229 [M − H]^−^; HR-ESI-MS *m*/*z* 229.0873 [M − H]^−^ (calcd for C_14_H_13_O_3_, 229.0870).

*Griseaketide C (**3**)*: Pale yellow oil; ${\left[\alpha \right]}_{D}^{18}$ +15.2 (*c* 0.31, MeOH); UV (MeOH) *λ*_max_ (log *ε*) 201 (4.47), 226 (4.45), 271 (3.39) nm; ^1^H NMR (CDCl_3_, 600 MHz) and ^13^C NMR (CD_3_OD, 150 MHz) data, see Table 2; negative ESI-MS *m*/*z* 231 [M − H]^−^; HR-ESI-MS *m*/*z* 231.1027 [M − H]^−^ (calcd for C_14_H_16_O_3_, 231.1027).

*Griseaketide D (**4**)*: Colorless amorphous powder; ${\left[\alpha \right]}_{D}^{18}$ –10.6 (*c* 0.31, MeOH); UV (MeOH) *λ*_max_ (log *ε*) 201 (3.44), 215 (3.07), 271 (2.54) nm; ^1^H NMR (CD_3_OD, 600 MHz) and ^13^C NMR (CD_3_OD, 150 MHz) data, see Table 2; positive ESI-MS *m*/*z* 273 [M + Na]^+^, 523 [2M + Na]^+^. HR-ESI-MS *m*/*z* 273.1096 [M + Na]^+^ (calcd for C_14_H_18_O_4_, 273.1097).

### 4.4. Nematicidal Activity 

The saprophytic nematode *C. elegans* was cultured on oatmeal medium (20 g of oatmeal in 80 mL of H_2_O) at 25 °C for 7 days. Then the cultured nematodes were separated from the culture medium using the Baerman funnel technique, and an aqueous suspension of the nematode was prepared as a working stock. Compounds **1** and **5**–**12** were dissolved in methanol and then diluted to different concentrations (400 and 200 ppm) with sterile water. The nematicidal activity against *C. elegans* was assayed according to the method based on references [31,32].

## Figures and Tables

**Table 1:** ^1^H and ^13^C NMR Data of Compounds **1** and **2** (*δ* in ppm, *J* in Hz).

No.	1 (in CDCl_3_)			2 (in CDCl_3_)		
	*δ*_H_	*δ*_C_	HMBC	*δ*_H_	*δ*_C_	HMBC
1	4.85, d (15.8)	64.3, t	2, 3, 7, 9	5.23, dd (11.9, 2.1)	71.2, t	2, 7, 8
	4.70, d (15.8)		2, 3, 7, 9	5.15, d (11.9)		2, 7, 8
2	-	121.9, s	-	-	125.2, s	-
3	-	150.8, s	-	-	150.3, s	-
4	6.65, d (8.7)	113.7, d	6	6.71, d (8.0)	114.5, d	2, 3, 6
5	7.11, t (7.5, overlap)	127.7, d	3, 4, 7	7.18, t (7.7)	129.4, d	3, 7
6	7.12, d (7.5, overlap)	118.4, d	2, 4, 7	6.73, d (7.5)	113.9, d	2, 4, 8
7	-	138.3, s	-	-	142.4, s	-
8	4.49, d (7.5)	69.2, d	7, 9, 10	5.73, brd (6.0)	84.4, d	9, 10
9	3.93, m	75.4, d	1, 11	6.25, dd (15.1, 6.8)	141.8, d	7, 8
10	2.98, dd (16.2, 4.7)	43.2, t	8, 9, 11	6.53, dd (15.1, 11.0)	129.0, d	8, 11, 12
	3.03, dd (16.2, 7.1)		-			
11	-	199.2, s	-	7.15, dd (15.7, 11.0)	142.4, d	9, 10, 13
12	6.24, dd (15.8, 1.4)	132.2, d	11, 14	6.21, d (15.7)	131.4, d	10, 14
13	6.99, dq (15.8, 6.8)	144.6, d	11, 14	-	198.8, s	-
14	1.94, dd (6.8, 1.4)	18.4, q	12, 13	2.28, s	27.3, q	12, 13

**Table 2:** ^1^H and ^13^C NMR Data of Compounds **3** and **4** (*δ* in ppm, *J* in Hz).

No.	3 (in CD_3_OD)			4 (in CD_3_OD)		
	*δ*_H_	*δ*_C_	HMBC	*δ*_H_	*δ*_C_	HMBC
1	5.09, dd (12.0, 2.6)	71.7, t	2, 7, 8	4.91, d (15.6)	65.8, t	2, 3, 7, 10
	4.98, brd (12.0)		2, 7, 8	4.50, d (15.6)		2, 3, 7
2	-	126.0, s	-	-	123.1, s	-
3	-	153.0, s	-	-	154.0, s	-
4	6.67, d (7.7)	115.1, d	1, 2, 3, 6	6.68, d (7.9)	114.6, d	3, 6
5	7.10, t (7.7)	130.3, d	3, 4, 7	7.08, t (7.9)	128.4, d	3, 6, 7
6	6.58, d (7.7)	113.8, d	2, 4, 8	6.85, d (7.5)	122.0, d	2, 4, 8
7	-	144.1, s	-	-	138.2, s	-
8	5.57, d (7.7)	86.6, d	7, 9	4.28, brs	67.5, d	2, 6, 7
9	5.71, dd (15.1, 7.7)	133.5, d	8, 11	2.06, m; 1.80, m	39.6, t	10, 11, 12
10	6.38, dd (15.1, 10.6)	132.8, d	8, 11, 12	3.63, brt (6.8)	76.9, d	8, 9
11	6.24, dd (15.2, 10.6)	129.3, d	10, 13	4.30, t (6.8)	71.2, d	13
12	5.81, dd (15.2, 6.2)	139.9, d	10, 13	5.52, dd (14.6, 6.8)	135.3, d	11, 14
13	4.29, dq (6.2, 6.4)	68.7, d	11, 14	5.73, dq (14.6, 6.1)	127.7, d	11, 14
14	1.24, d (6.4)	23.5, q	12, 13	1.71, d (6.1)	17.9, q	12, 13
